# Draft Genome Sequence of Serratia marcescens Strain ZZCCN01, Isolated from the Cardiac Blood of a Beef Cow

**DOI:** 10.1128/MRA.00728-20

**Published:** 2020-12-03

**Authors:** Yu-Long Zhang, Fang-Yuan Huang, Cai-Xia Yao, Dong-Jie Cai, Zhi-Sheng Wang, Zhi-Cai Zuo

**Affiliations:** a Key Laboratory of Animal Disease and Human Health of Sichuan Province, College of Veterinary Medicine, Sichuan Agricultural University, Chengdu, Sichuan, China; b Animal Nutrition Institute, Sichuan Agricultural University, Chengdu, Sichuan, China; University of Maryland School of Medicine

## Abstract

Serratia marcescens strain ZZCCN01 was isolated from the cardiac blood of a dead beef cow with a lung infection and a foam-like secretion from the nostril. Here, we introduce the 5.1-Mb draft genome sequence, which comprises 105 scaffolds, and the corresponding annotation.

## ANNOUNCEMENT

Serratia marcescens is a Gram-negative, clinically opportunistic pathogen. It can cause infection and sepsis targeting the lung and urinary tract when the immune system is impaired (1, 2), as well as systemic infections and death (3).

Serratia marcescens strains were obtained from the blood samples from a dead beef cow through pathological dissection. Cardiac blood was collected from the beef cow aseptically with vacuum blood collection tubes, preserved in ice-cold condition, and transported to the laboratory. Samples were streaked onto lysogeny broth agar and incubated overnight at 37°C for 18 h. Single colonies were utilized to inoculate lysogeny broth, from which genomic DNA was isolated with the TIANamp bacterial DNA kit (Tiangen Biotech, Beijing, China).

For sequencing, extracted DNA was utilized to prepare shotgun libraries for Illumina sequencing following the instructions of the manufacturer (Illumina, San Diego, CA, USA). A NEBNext Ultra DNA library prep kit was used to make the Illumina sequencing library (New England Biolabs, Beverly, MA, USA). Sequencing by an Illumina HiSeq instrument resulted in 9,683,553-bp paired-end Illumina reads (150 bp). The reads were trimmed using Trimmomatic version 3.5.0 with default settings. The initial hybrid *de novo* assembly was performed using SPAdes software version 3.9.0 with default settings (4, 5) and resulted in 105 scaffolds. The correction was performed using PrlnSeS-G version 1.0.0 with default settings (6). The genome of Serratia marcescens strain ZZCCN01 had an overall G+C content of 59%, and the *N*_50_ value of the contigs from the genome assembly was 150,116 bp. The genome sequence was annotated using the NCBI Prokaryotic Genome Annotation Pipeline (PGAP) (https://www.ncbi.nlm.nih.gov/genome/annotation_prok/), and the results showed that it has 4,910 genes, 4,795 coding DNA sequences (CDSs), 80 tRNAs, 10 rRNAs, and 25 noncoding RNAs (ncRNAs).

The genome sequence was annotated using the Virulence Factor Database (http://www.mgc.ac.cn/VFs), which yielded 1,339 virulence factors or virulence factor-related genes, such as SLS, which lyses a wide variety of eukaryotic cells, including myocardial cells, kidney cells, platelets, lymphocytes, and neutrophils (7); *htpB*, whose product, hsp60, was involved in apoptosis (8); *mip*, the macrophage infectivity potential gene (9); *pglJ*, which participates in the colonization of intestinal niches in a wide variety of hosts (10); and *farA*, which mediates the resistance to antimicrobial long-chain fatty acids.

The drug resistance functions were annotated by the Comprehensive Antibiotic Resistance Database (https://card.mcmaster.ca/), which yielded 4,627 antibiotic resistance-associated genes, such as *OXA-58*, a class D β-lactamase (11); *folP*, a sulfonamide resistance gene (12); *AAC6*, an aminoglycoside resistance gene (13); and *fsr*, a fosmidomycin resistance gene (14). ZZCCN01 was found to contain the small multidrug resistance protein (AcrAB family) and multidrug efflux pumps (15), indicating its potential to become a multidrug-resistant strain.

Serratia marcescens infection is a global public health problem. ZZCCN01 was isolated from the cardiac blood of a beef cow. Therefore, it is of great significance to determine the genomic sequence of ZZCCN01 for better understanding of the infection mechanisms of Serratia marcescens in humans and animals.

### Data availability.

This whole-genome shotgun sequencing project has been deposited at DDBJ/EMBL/GenBank under the accession no. JABTVN000000000.1. The associated BioProject, SRA, and BioSample accession numbers are PRJNA626063, SRP257993, and SAMN14615110, respectively.
